# Ecto- and endo-parasitic monogeneans (Platyhelminthes) on cultured freshwater exotic fish species in the state of Morelos, South-Central Mexico

**DOI:** 10.3897/zookeys.776.26149

**Published:** 2018-07-26

**Authors:** Edgar F. Mendoza-Franco, Juan Manuel Caspeta-Mandujano, Marina Tapia Osorio

**Affiliations:** 1 Instituto de Ecología, Pesquerías y Oceanografía del Golfo de México (EPOMEX), Universidad Autónoma de Campeche, Campeche, México Universidad Autónoma de Campeche Campeche Mexico; 2 Facultad de Ciencias Biológicas y Centro de Investigaciones Biológicas, Universidad Autónoma del Estado de Morelos, Cuernavaca, Morelos, Mexico Universidad Autónoma del Estado de Morelos Morelos Mexico; 3 Centro Nacional de Servicios de Constatación en Salud Animal Jiutepec, Morelos, México Centro Nacional de Servicios de Constatación en Salud Animal Jiutepec Morelos Mexico

**Keywords:** characids, cichlids, cyprinids, fish introductions, loricariids, Monogenea, Morelos state, non-native ornamental fish, osphronemids, pangasids, parasites, poeciliids, quarantine, tilapia

## Abstract

An extensive parasitological study of 365 freshwater exotic fish specimens belonging to 13 species of seven families (Cichlidae, Cyprinidae, Osphronemidae, Pangasidae, Poeciliidae, Characidae, and Loricariidae) collected from 31 Aquaculture Production Units (APU) from Central Mexico revealed the occurrence of 29 ecto- and endo-parasitic monogeneans found on gills and stomachs: *Cichlidogyrussclerosus*, *C.thurstonae*, *C.tilapiae*, *Cichlidogyrus* sp. 1, *Cichlidogyrus* sp. 2, *Enterogyruscoronatus*, *E.malmbergi*, *Gusseviaspiralocirra*, *Sciadicleithrumiphthimum*, *Sciadicleithrum* sp., *Scutogyruslongicornis* (all Dactylogyridae), *Gyrodactyluscichlidarum*, and *G.yacatli* (Gyrodactylidae) on *Oreochromisniloticus*, *Pterophyllumscalare* and *Hemichromis* sp. (Cichlidae); *Dactylogyrusbaueri*, *D.formosus*, *D.intermedius*, *D.vastator*, *D.extensus*, *Dactylogyrus* sp. (all Dactylogyridae), and *G.kobayashii* on *Carassiusauratus*, *Cyprinuscarpio* and *Ctenopharyngodonidella* (Cyprinidae); *Trianchoratusacleithrium* and *T.trichogasterium* (Dactylogyridae) on *Trichogastertrichopterus* (Osphronemidae); *Thaparocleiduscaecus*, *T.siamensis* (Dactylogyridae), and Dactylogyridae sp. on *Pangasianodonhypophthalmus* (Pangasidae); *G.poeciliae* on *Poeciliareticulata* (Poeciliidae); *Diaphorocleidusarmillatus* (Dactylogyridae) on *Gymnocorymbusternetzy* (Characidae); *Unilatusunilatus* (Dactylogyridae) and Gyrodactylidae sp. on *Hypostomus* sp. (Loricariidae). The paramount importance of the establishment of these monogeneans due to the importation/exportation of non-native ornamental and other exotic host fish species cultured for food in Mexico is briefly discussed. Quarantine is recommended for all transferred host species.

## Introduction

At a global level, increasing attention is being paid to generate useful ecological indicators that favor invasiveness and geographic range expansion by introduced species (Lavergne and Molofsky 2007, Blackburn and Ewen 2017). Conjointly, introductions of species are rising sharply because of increased trade, transport, travel, and tourism associated with globalization (IPPC Secretariat 2005). Within this context, trade of the non-native ornamental fish industry and/or fish farms for food production, has been the main cause of introductions of fish and their parasites around the world (Barroso de Magalhães and Jacobi 2013, Mendoza et al. 2015). Furthermore, the same industries pose a growing threat to native wildlife if non-native fishes are later released into the wild (see Mendoza-Franco et al. 2012). Culture of non-native ornamental and food fishes represents major activities in the state of Morelos (south-central Mexico) since these fishes are commercially distributed within and outside of Mexico in large quantities (Martínez et al. 2010).

Although non-native aquatic organisms are important to Morelos aquaculture and the economy of the state of Morelos, the aquaculture industry should be made aware of the considerable local, state, and national concern over the potential ecological or economic problems arising from non-native fish introductions and their parasites in natural environments (i.e., parasite transfer and/or fish competition with native species) (Barroso de Magalhães and Jacobi 2013). Recently, a total of 44 helminth species on introduced freshwater fishes were listed for Mexico, of which five are invasive species, i.e., *Cichlidogyrussclerosus* Paperna & Thurston, 1969 *Dactylogyrusextensus* Mueller & Van Cleave, 1932 and *Gyrodactyluscichlidarum* (Paperna 1968) García-Vasquez & Hansen, 2007 (Monogenea); *Centrocestusformosanus* (Nishigori 1924) Price, 1932 (Digenea) and *Schyzocotyleacheilognathi* Yamaguti, 1934 (Cestoda), all of them introduced with their Asian and African hosts (Tapia Osorio et al. 2014). The present study was conducted to identify the most common ecto- and endo-parasitic monogeneans inhabiting commercially important ornamental and/or food fish species that have been imported into Mexico.

## Materials and methods

Ornamental fish species were collected from 2010 to 2014 from different municipalities (Axochiapan, Ayala, Cuautla, Jiutepec, Jojutla, Tlaltizapan, Tlaquiltenango, Xochitepec, and Zacatepec) located in the state of Morelos. Live fish were examined thoroughly externally under a stereo-microscope before opening the visceral cavity. Fish were sacrificed by puncturing the brain region and the gills of each ﬁsh were removed and placed in vials containing hot 4–5% formalin solution to ﬁx any of the ectoparasites that might be present and labeled with data of each collection site. The internal cavity of each fish was exposed by an incision made along the venter from the anus to mouth. The entire alimentary canal was removed; the interior of the gut was thoroughly examined in situ, then placed in a Petri dish containing hot formalin solution 4–5%, where it was searched for monogeneans (Salgado-Maldonado et al. 2014). Subsequently, all monogeneans specimens were isolated and stained with Gomori’s trichrome and mounted in Canada balsam. In addition, some specimens were mounted in a mixture of lactic-acid (LA) and glycerin- ammonium picrate (GAP) and then remounted in Canada balsam as permanent preparations (Mendoza-Franco et al. 2013). Parasite identifications were made using a Leica microscope DM2500 with Nomarski interference contrast and based on descriptions provided in the following references: García-Vásquez et al. 2007, 2015, Jogunoori et al. 2004, Kritsky et al. 1989, Lim 1996, Mendoza-Palmero et al. 2012, Pariselle and Euzet 1995, Yamaguti 1963. Reference specimens were deposited in the National Helminthological Collection of Mexico (**CNHE**). Prevalence (percent of hosts infected), mean abundance (mean number of parasites per examined fish), and intensity range for each monogenean species follows Bush et al. (1997). Host species and common names follow those in the FishBase (Froese and Pauly 2017).

## Results

**Figure 1.**
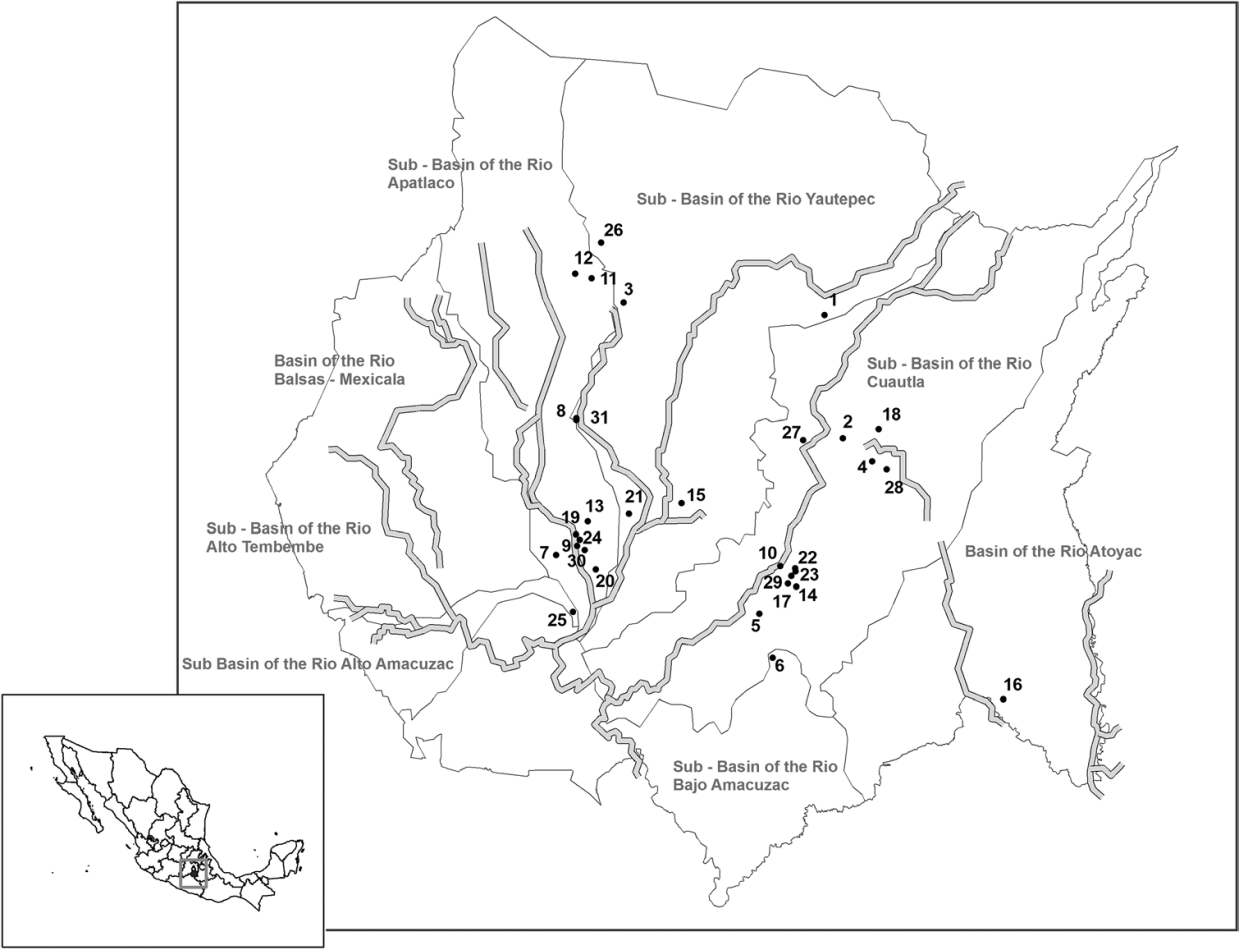
Map of the state of Morelos, Mexico showing position of each APU: **1** 7 Hermanos (18°51'49.82132"N; 98°58'01.20211"W**2** Acuícola Ayala (18°45'11.59525"N; 98°56'58.87989"W) **3** Acuícola de Jiutepec (18°52'29.84116"N; 99°09'24.49751"W) **4** Acuícola Jaloxtoc (18°43'56.72740"N; 98°55'20.14003"W) **5** Adilene Marisol (18°35'43.94208"N; 99°01'43.49419"W) **6** Agua Fría (18°33'22.41096"N; 99°00'57.44948"W) **7** Aquafish (18°38'53.20757"N; 99°13'13.80019"W) **8** Betta Fish (18°46'15.00012"N; 99°12'05.44263"W) **9** Centro Zacatepec (18°39'22.70079"N; 99°12'02.36030"W) **10** Centro de Acopio La Perla (18°38'18.23968"N; 99°00'32.15165"W) **11** Consorcio Lugo-Galeana (18°53'48.34681"N; 99°11'13.92251"W) **12** El Chino (18°54'03.35178"N; 99°12'10.27438"W) **13** El Cifón (18°40'42.68111"N; 99°11'26.16448"W) **14** El Invernadero (18°37'11.86468"N; 98°59'37.85120"W) **15** Exopez (18°41'41.78829"N; 99°06'07.81780"W) **16** Granja Acuícola Foras (18°31'07.09460"N; 98°47'54.39963"W); **17.** Grupo Carsal (18°37'21.23567"N; 99°00'05.49462"W) **18** Huertas de Cuatla (18°45'41.45252"N; 98°54'57.10516"W) **19** Jesús Madariaga (18°39'59.91903"N; 99°12'05.85187"W) **20** La Buena Fortuna (18°38'07.31312"N; 99°10'58.58424"W) **21** La Cascada (18°41'06.91860"N; 99°09'05.97650"W) **22** Linda Vista (18°38'11.27728"N; 98°59'41.36454"W) **23** Los Huajes (18°38'01.06064"N; 98°59'39.86312"W) **24** Maleny (18°39'43.43675"N; 99°11'52.86078"W) **25** Maricultura Argos (18°35'50.18775"N; 99°12'16.44262"W) **26** Olascoaga (18°55'43.39346"N; 99°10'40.92078"W) **27** Ornapez (18°45'06.02177"N; 98°59'14.37030"W) **28** Platanar (18°43'30.25259"N; 98°54'30.22690"W) **29** Pliego (18°37'45.93123"N; 98°59'53.99321"W) **30** San Tilapia (18°39'09.51796"N; 99°11'36.53955"W) **31** Tropipez (18°46'10.83544"N; 99°12'05.47184"W).

A total of 365 fish specimens of 13 species belonging to 7 families was examined for monogeneans: Cichlidae, Characidae, Cyprinidae, Loricariidae, Osphronemidae, Pangasidae, and Poeciliidae. Twenty-nine monogenean species infecting gills and/or stomachs were identified from hosts species of all families mentioned above from a total of 31 Aquaculture Production Units (APU) from different municipalities located in the state of Morelos (see Table 1 and Figure 1). The prevalence, mean abundance, and mean intensity of infections at each APU of individual species from different hosts are provided in Tables 2–4.

**Table 1.** Ecto- and endo-parasitic monogeneans (Platyhelminthes) on cultured exotic fish from several Aquaculture Production Units (APU) in the state of Morelos, South-Central Mexico.

Host species/Family	Monogeneans/CNHE	APU	Municipalities
*Oreochromisniloticus* (**Cichlidae**)	*Cichlidogyrussclerosus*^†^ /*10743*	Acuícola Jaloxtoc El Cifón 7 Hermanos La cascada Acuícola Ayala Maricultura Argos	Ayala Zacatepec Cuautla Tlaltizapan Ayala Zacatepec
	*Cichlidogyrusthurstonae*^†^ /*10744*	La Cascada	Tlaltizapan
	*Cichlidogyrustilapiae*^†^ /*10745*	Acuícola Ayala	Ayala
		Maricultura Argos	Zacatepec
* Oreochromis niloticus *	*Cichlidogyrus sp. 1 ^†^ /*10746* *Cichlidogyrus sp. 2 ^†^ /*10747*	Acuícola Ayala	Ayala
*Hemichromis* sp.	*Enterogyruscoronatus*^‡^ /*10748*	Maleny	Zacatepec
* Oreochromis niloticus *	*Enterogyrusmalmbergi*^‡^ /*10749-10750*	Acuícola Ayala Adilene Marisol San Tilapia Acuícola de Jiutepec Pliego	Ayala Ayala Tlaltizapan Juitipec Ayala
*Oreochromis* sp.	*Enterogyrusmalmbergi*^‡^ /*10751*	San Tilapia La buena Fortuna	Tlaltizapan Jojutla
* Oreochromis niloticus *	*Gyrodactyluscichlidarum*^£^ /*10756*	Acuícola Jaloxtoc Centro Zacatepec	Ayala Zacatepec
	*Gyrodactylusyacatli^£^ /10757*	Centro Zacatepec	Zacatepec
* Pterophyllum scalare *	*Gusseviaspiralocirra^†^ /*10752* *Sciadicleithrumiphthimum^†^ /*10753* *Sciadicleithrum sp. ^†^ /*10754*	Jesús Madariaga	Zacatepec
		El Chino	Juitepec
		Olascoaga	Juitepec
* Oreochromis niloticus *	*Scutogyruslongicornis*^†^ /*10755*	La Cascada	Tlaltizapan
*Carassiusauratus* (**Cyprinidae**)	*Dactylogyrusbaueri^†^ /*10758* *Dactylogyrusformosus^†^ /*10759* *Dactylogyrusintermedius^†^ /*10760Dactylogyrusvastator*^†^ /*10761-10762*	Centro de acopio La Perla El Invernadero Los Huajes Linda Vista Platanar Grupo Carsal	Tlaltizapan Ayala Ayala Ayala Ayala Ayala
	*Gyrodactyluskobayashii^£^ /*10765-10767*	Linda Vista Los Huajes Grupo Carsal El Invernadero	Ayala Ayala Ayala Ayala
* Cyprinus carpio *	*Dactylogyrusextensus^†^ /*10763*	Ornapez	Ayala
* Ctenopharyngodon idella *	*Dactylogyrus sp. ^†^ /*10764*	Centro Zacatepec	Zacatepec
*Trichogastertrichopterus* (**Osphronemidae)**	*Trianchoratusacleithrium^†^ /*10768*	Consorcio Lugo-Galeana Granja Acuícola Foras	Jiutepec Axochiapan
	*Trianchoratustrichogasterium^†^ /*10769*	Consorcio Lugo-Galeana	Jiutepec
* Pangasianodon hypophthalmus * **(Pangasidae)**	*Thaparocleiduscaecus^†^ /*10770* *Thaparocleidussiamensis^†^ /*10771-10772*	Betta Fish Betta Fish La buena Fortuna	Xochitepec Xochitepec Jojutla
	*Dactylogyridae sp. ^†^	La buena Fortuna	Jojutla
* Poecilia reticulata * **(Poeciliidae)**	*Gyrodactyluspoeciliae^£^ /10773	Huertas de Cuatla Exopez Agua Fría	Ayala Tlaltizapan Tlaquiltenengo
* Gymnocorymbus ternetzy * **(Characidae)**	*Diaphorocleidusarmillatus^†^ /*10774-10775*	Aquafish Tropipez	Zacatepec
*Hypostomus* sp. **(Loricariidae)**	*Gyrodactylidae sp. ^£^ /10777 *Unilatusunilatus^†^ /*10776*	Consorcio Lugo-Galeana	Jiutepec
		Consorcio Lugo-Galeana	Jiutepec

* = new record in Mexico. Site of infection on host: † = gills lamellae; ‡ = stomach; £ = fins.

## Discussion

Currently, 31 species of exotic monogeneans have been registered in the state of Morelos due to the introduction of their hosts that are cultured either for food or aquariums (present data; Caspeta-Mandujano et al. 2009). This current study on cultured exotic fish species revealed that cichlids (i.e., species of *Oreochromis*, *Hemichromis*, and *Pterophyllum*), harbored the highest number of monogeneans (14 species) followed by cyprinids with seven species of which *Dactylogyrusbaueri* Gussev, 1955, *Dactylogyrusformosus* Kulwieć, 1927, *Dactylogyrusintermedius* Wegener, 1909, and *Gyrodactyluskobayashii* Hukuda, 1940 are new geographical records in Mexico (see Tables 1 and 3). Despite the great number of parasitological studies on native and/or introduced species of Cichlidae in Mexico (Vidal-Martínez et al. 2001), studies on the parasite fauna of other exotic freshwater fishes, especially on their monogeneans, are relatively scarce. Exceptionally, there have been many reports of species of *Cichlidogyrus* on species of *Oreochromis* (often called tilapia) (see Kritsky et al. 1994, Jiménez-García et al. 2001). Even so, intensity of infection is comparatively high as well as the number of new records of these monogeneans, the latter which continues to grow each year (see Table 3, Mendoza-Franco et al. 2015b). In the present study, the angelfish *P.scalare* (Schultze) and *Hemichromis* sp. were studied for the first time and are shown to be parasitized with *G.spiralocirra* Kohn & Paperna, 1964, *S.iphthimum* Kritsky, Thatcher & Boeger, 1989, *Sciadicleithrum* sp. (new geographical records), and *E.coronatus* Pariselle, Lambert & Euzet, 1991.

**Table 2.** Parameters of infection of monogeneans on cichlids (APU: Aquaculture Production Unit; P%: Prevalence; MA: mean abundance; RI: range of infection; MI: mean intensity; IH: infected hosts).

APU	Hosts	Monogeneans	Inds.	P%	MA	RI	MI	IH
Maleny	*Hemichromis* sp.	* Enterogyrus coronatus *	36	50	5.14	1–13	3.6	10/20
7 hermanos	* Oreochromis niloticus *	* Cichlidogyrus sclerosus *	12	57	1.71	2–4	3.0	4/7
Acuícola de Jiutepec	* Oreochromis niloticus *	* Enterogyrus malmbergi *	18	50	2.57	2–5	3.6	5/10
Acuícola Jaloxtoc	* Oreochromis niloticus *	* Gyrodactylus cichlidarum *	18	20	2.57	18	18	1/5
	* Oreochromis niloticus *	* Cichlidogyrus sclerosus *	13	100	2.60	1–7	2.6	5/5
Adilene Marisol	* Oreochromis niloticus *	* Enterogyrus malmbergi *	53	100	7.57	2–13	5.3	10/10
Centro Zacatepec	* Oreochromis niloticus *	* Gyrodactylus yacatli *	15	10	2.14	15	15	1/10
El Cifón	* Oreochromis niloticus *	* Cichlidogyrus sclerosus *	7	40	1.00	3–4	3.5	2/5
Acuícola Ayala	* Oreochromis niloticus *	* Cichlidogyrus tilapiae *	159	100	22.71	3–37	15.9	10/10
	* Oreochromis niloticus *	* Enterogyrus malmbergi *	6	50	0.86	1–2	1.2	5/10
	* Oreochromis niloticus *	* Enterogyrus malmbergi *	1	10	0.14	1	1.0	1/10
Pliego	* Oreochromis niloticus *	* Enterogyrus malmbergi *	2	25	0.29	2	2.0	1/4
San Tilapia	* Oreochromis niloticus *	* Enterogyrus malmbergi *	34	100	4.86	1–17	8.5	4/4
	*Oreochromis* sp.	* Enterogyrus malmbergi *	23	60	3.29	1–7	3.83	6/10
La Buena Fortuna	*Oreochromis* sp.	* Enterogyrus malmbergi *	76	76.9	10.86	2–19	7.6	10/13
Jesús Madariaga	* Pterophyllum scalare *	* Gussevia spiralocirra *	5	10	0.71	5	5.0	1/10
El Chino	* Pterophyllum scalare *	*Sciadicleithrum* spp.	6	83.3	1.00	1–2	1.2	5/6
Olascoaga	* Pterophyllum scalare *	*Sciadicleithrum* sp.	9	75	1.29	1–4	3.0	3/4

**Table 3.** Parameters of infection of monogeneans on hosts of the Cyprinidae (APU: Aquaculture Production Unit; P%: Prevalence; MA: mean abundance; RI: range of infection; MI: mean intensity; IH: infected hosts).

APU	Hosts	Monogeneans	Inds.	P%	MA	RI	MI	IH
Consorcio Lugo-Galeana	* Carassius auratus *	*Dactylogyrus* sp.	520	100	52.0	13–154	86.7	10/10
El invernadero	* Carassius auratus *	* Gyrodactylus kobayashii *	525	100	87.5	5–314	87.5	6/6
	* Carassius auratus *	* Dactylogyrus formosus *	1	17	0.17	1–8	1.0	1/6
Grupo Carsal	* Carassius auratus *	* Gyrodactylus kobayashii *	28	100	20	0.3–54	26.7	3/3
	* Carassius auratus *	* Dactylogyrus vastator *	5	33	1.7	5	5.0	1/3
Linda vista	* Carassius auratus *	* Gyrodactylus kobayashii *	12	20	1.2	2–10	6	2/10
	* Carassius auratus *	* Dactylogyrus vastator *	3	10	0.3	3	3.0	1/10
Los Huajes	* Carassius auratus *	* Dactylogyrus baueri *	1	20	0.2	1	1.0	5/5
	* Carassius auratus *	*Dactylogyrus* spp.	38	100	7.6	2–25	7.6	5/5
	* Carassius auratus *	* Gyrodactylus kobayashii *	102	100	20.4	2–58	20.4	5/5
Centro Zacatepec	* Ctenopharyngodon idella *	*Dactylogyrus* sp.	100	14	14.3	100	100.0	1/7
Ornapez	* Cyprinus carpio *	* Dactylogyrus extensus *	5	20	0.5	2–3	2.5	2/10

Monogeneans usually exhibit high host specificity in comparison with other parasite groups, parasitizing a single or few closely related host species. The only zoogeographic range expansion of exotic monogeneans on native hosts is the discovery of species of *Cichlidogyrus* and *G.cichlidarum* from tilapia on native cichlids and poeciliids, respectively, in natural environments of Mexico (Jiménez-García et al. 2001, García-Vásquez et al. 2007, 2017). The present study revealed the highest intensity of infection with *G.cichlidarum* (identified as a tilapia pathogen by García-Vásquez et al. 2017) and *Cichlidogyrus* spp. on *Oreochromis* spp. (see Table 2). Therefore, preventing escape of these tilapia from culture systems due to their monogeneans’ ability to infest and persist on other non- or related wild fish is urgently required. Another example of the persistence of monogeneans is seen with the dactylogyrid *Urocleidoidesvaginoclaustrum* Jogunoori, Kritsky & Venkatanarasaiah 2004. This monogenean was originally described from fishes introduced to India via the aquarium trade. Its type host, the green swordtail *Xiphophorushellerii* (Heckel) (Poeciliidae), is naturally distributed in southern Mexico and Central America, where the native profundulid *Profunduluslabialis* (Günther) also hosts *U.vaginoclaustrum*. The problem is that *X.hellerii* has been artificially introduced along with *U.vaginoclaustrum* to other hydrological systems such as India and northern Mexico (Jogunoori et al. 2004, Mendoza-Palmero and Aguilar-Aguilar 2008, Mendoza-Franco et al. 2015a) from which other cyprinodontiform hosts could potentially become infected with this parasite. Additionally, in the present study the black tetra *G.ternetzi* (Boulenger) (Characidae) was studied for the first time and is revealed to be highly infested with *D.armillatus* Jogunoori, Kritsky & Venkatanarasaiah, 2004 (Dactylogyridae) (see Table 4). *Gymnocorymbusternetzi* is native to South America and has been introduced via the aquarium trade to India and Mexico. Currently, there are nine species of *Diaphorocleidus* dispersed on native bryconid and characid (Characiformes) hosts in the neotropics (South and Central America) (Santos et al. 2018). The transfer and/or evidence of extensive cryptic speciation of other monogenean groups from exotic to native or vice versa on closely related hosts in Mexico remains unknown, but that potential exists.

Similarly to the introduced tilapia in Mexico, cyprinids (i.e., *C.idella*) are also widely distributed in the country including habitats located within areas protected for conservation (see Salgado Maldonado et al. 2014). These fishes were introduced to Central America (i.e., Mexico and Honduras) for aquaculture purposes from 1965-1980s (Salgado-Maldonado and Rubio-Godoy 2014, Salgado-Maldonado et al. 2015) and the presence of species of *Dactylogyrus* and *G.kobayashii* (see Table 1, 3) in Morelos might be originally related to these introductions. Poeciliids (known as guppies, mollies, platies, and swordtails) have been studied for ectoparasitic monogeneans in Mexico and mainly gyrodactylids have been reported on the skin and/or gills on these fishes (García-Vásquez et al. 2015). Currently, there are 11 gyrodactylid species described and/or reported from poeciliids. Only species of *Urocleidoides* (Dactylogyridae) have been reported on the gills of the poeciliids of the two-spot livebearers *Pseudoxiphophorusbimaculata* (Heckel), *X.hellerii*, and *Poeciliopsisretropinna* (Regan) from Mexico and Panama (Mendoza-Franco et al. 2015). In the present study, *G.poeciliae* Harris & Cable, 2000 was found for the first time on the guppy *Poeciliareticulata* Peters from Mexico (see Tables 1, 4). This monogenean species has been reported on *Poeciliacaucana* (Steindachner) and *P.reticulata* from their natural ranges of distribution (Venezuela and Trinidad, respectively). Among all species of *Gyrodactylus* mentioned above, only *G.bullatarudis* Turnbull, 1956 and *G.turnbulli* Harris, 1986 have been reported on six poeciliid host species (*Gambusiaholbrooki* Girard, *Poeciliasphenops* Valenciennes, *P.reticulata*, *P.bimaculata*, *Poeciliopsis* sp., and *X.hellerii*) from Mexico, Canada, Costa Rica, Peru, Trinidad, Australia, and Singapore (see García-Vásquez et al. 2015). Given the low host specificity of both gyrodactylid species and the invasive characteristic of poeciliids, the potential transfer of these gyrodactylids to native poeciliids and other ecologically-associated hosts in Mexico is high (see García-Vásquez et al. 2017, Mendoza-Franco et al. 2015).

The African tilapia (Cichlidae) and the Asian catfish (Pangasiidae) are both freshwater whitefish aquaculture species that potentially compete for similar markets. In fact, in 2013 Mexico was recognized as the second largest importer of pangasius fillet in the world (Martínez et al. 2016). No analysis concerning the environmental impact of the introduction of these latter fishes and their parasites from Vietnam into Mexican aquaculture and/or in wild habitats (Martínez et al. 2016) has been made. *Pangasianodonhypophthalmus* (Sauvage) was studied for the first time in the present study and it revealed to be parasitized with three monogenean species: *Thaparocleiduscaecus* (Mizelle & Kritsky, 1969) Lim 1996, *T.siamensis* (Lim 1990) Lim, 1996, and Dactylogyridae sp. (Table 4). Finally, Loricariids, otherwise known as plecos (species of *Hypostomus*) are very popular ornamental freshwater fish naturally found in tropical South America, Panama, and Costa Rica. In Mexico, *Hypostomusplecostomus* (L.) was introduced into the Balsas Basin (see geographic position in Figure 1) to control macrophytes and algae, and are now established in multiple water bodies (Ramírez-Morales and Ayala-Pérez 2009). The only report of a gill monogenean species on an introduced pleco to Mexico is that of *Heteropriapulus* sp. (Dactylogyridae) on the Amazon sailfin *Pterygoplichthyspardalis* Castelnau from the Reserva de la Biosfera Montes Azules (BRMA) in the state of Chiapas (Mendoza-Franco et al. 2012). The present study provides two new monogenean records for Mexico, Gyrodactylidae sp. and *Unilatusunilatus*, the latter belonging to the Dactylogyridae which was previously reported on the snow pleco *P.anisitsi* Eigenmann and Kennedy and on *Plecostomus* sp., from Brazil and Peru, respectively (Mendoza-Palmero et al. 2012).

**Table 4.** Parameters of infection of monogeneans on characids, loricariids, osphronemids, pangasids, and poeciliids (APU: Aquaculture Production Unit; P%: Prevalence; MA: mean abundance; RI: range of infection; MI: mean intensity; IH: infected hosts).

APU	Host	Monogeneans	Inds.	P%	MA	RI	MI	IH
Aquafish	* Gymnocorymbus ternetzi *	* Diaphorocleidus armillatus *	131	100	13.1	2–24	13.1	10/10
Tropipez	* Gymnocorymbus ternetzi *	* Diaphorocleidus armillatus *	698	100	69.8	7–217	69.8	10/10
Consorcio Lugo-Galeana	*Hypostomus* sp.	* Unilatus unilatus *	15	60	1.5	1–11	2.5	6/10
	*Hypostomus* sp.	*Gyrodactylus* sp.	14	60	1.4	1–8	2.3	6/10
	* Trichogaster trichopterus *	*Trianchoratus* spp.	80	75	20	03–54	26.7	3/4
	* Trichogaster trichopterus *	* Trianchoratus trichogasterium *	250	80	25	16–61	31.3	8/10
Granja Acuícola Foras	* Trichogaster trichopterus *	* Trianchoratus trichogasterium *	564	90	56.4	1–262	62.7	9/10
Betta fish	* Pangasianodon hypophthalmus *	*Thaparocleidus* spp.	536	40	26.8	1–125	67.0	8/20
La Buena Fortuna	* Pangasianodon hypophthalmus *	* Thaparocleidus siamensis *	1000	100	200	130–300	200.0	5/5
	* Pangasianodon hypophthalmus *	Dactylogyridae sp.	10400	100	2080	1000–3000	1733.3	5/5
Exopez	* Poecilia reticulata *	* Gyrodactylus poeciliae *	4	33	0.67	2	2.0	2/6
Agua fría	* Poecilia reticulata *	* Gyrodactylus poeciliae *	75	90	7.5	1–37	8.3	9/10
Huertas de Cuautla	* Poecilia reticulata *	* Gyrodactylus poeciliae *	1	12.5	0.125	1	1.0	1/8

The fish examined in the present study are ornamental and/or for food production that are commercialized in Mexico. Results clearly show that importation of these fish can carry several monogeneans, both ecto- and endo-parasitic species, which could infect other related fish in systems they invade. Therefore, determining the occurrence of parasitic species will help provide better aquaculture conditions and will help to solve some of the problems faced by fish farmers. In the literature, there are a number of reports dealing with the introduction of parasites by ornamental fish from which the consequences of parasite introduction can be detrimental to native fish. For example, epizootics that may lead to extensive mortality (i.e., *D.vastator* on cyprinids, see Cone 1999) as shown for several species of monogeneans introduced into farms or aquariums, and from there to natural populations (Bakke et al. 2002, 2007; García-Vásquez et al. 2017). In addition to the identification of invasive host fish species, it is recommended that all freshwater fish imported into the country for food (farmed) or ornamental purposes must comply, at least, with quarantine regulations.
